# Evolution of *RAD*- and *DIV*-Like Genes in Plants

**DOI:** 10.3390/ijms18091961

**Published:** 2017-09-13

**Authors:** Ao Gao, Jingbo Zhang, Wenheng Zhang

**Affiliations:** Department of Biology, Virginia Commonwealth University, 1000 West Cary Street, Richmond, VA 23284, USA; gaoa2@mymail.vcu.edu (A.G.); jzhang5@vcu.edu (J.Z.)

**Keywords:** *RADIALIS*-like genes, *DIVIRICATA*-like genes, gene duplication, angiosperms, phylogeny, antagonism of proteins, MYB gene family

## Abstract

Developmental genetic studies of *Antirrhinum majus* demonstrated that two transcription factors from the MYB gene family, RADIALIS (RAD) and DIVIRICATA (DIV), interact through antagonism to regulate floral dorsoventral asymmetry. Interestingly, similar antagonistic interaction found among proteins of FSM1 (RAD-like) and MYBI (DIV-like) in *Solanum lycopersicum* is involved in fruit development. Here, we report the reconstruction of the phylogeny of I-box-like and R-R-type clades, where *RAD*- and *DIV*-like genes belong, respectively. We also examined the homology of these antagonistic MYB proteins using these phylogenies. The results show that there are likely three paralogs of *RAD*-/*I-box*-like genes, RAD1, RAD2, and RAD3, which originated in the common ancestor of the core eudicots. In contrast, *R-R*-type sequences fall into two major clades, RR1 and RR2, the result of gene duplication in the common ancestor of both monocots and dicots. RR1 was divided into clades RR1A, RR1B, and RR1C, while RR2 was divided into clades RR2A/DIV1, RR2B/DIV2, and RR2C/DIV3. We demonstrate that among similar antagonistic interactions in *An. Majus* and *So. lycopersicum*, *RAD*-like genes originate from the RAD2 clade, while *DIV*-like genes originate from distantly related paralogs of the R-R-type lineage. The phylogenetic analyses of these two MYB clades lay the foundation for future comparative studies including testing the evolution of the antagonistic relationship of proteins.

## 1. Introduction

The MYB gene family comprises three members, A-, B- and c-MYB [1,2], found in many vertebrates, that are involved in the regulation of cell proliferation, differentiation, and apoptosis [3]. Homologs of *MYB* genes have also been identified in insects, fungi, and slime molds [4]. The first plant *MYB* gene, *C1*, was isolated from *Zea mays*, and it encodes a c-MYB-like transcription factor involved in anthocyanin biosynthesis [5]. Plant MYB proteins were found to be involved in the regulation of many developmental processes including the biosynthesis of anthocyanin and flavonoids, trichome differentiation, the determination of cell shapes, and the regulation of cell proliferation and cell cycles [5,6,7,8,9].

In plants, the *MYB* genes have also been found in the regulation of the development of floral symmetry in the Lamiales [10]. In the zygomorphic flowers of *Antirrhinum majus* L., the two dorsal petals are significantly enlarged compared to the lateral and ventral petals, and the single dorsal stamen is aborted [11]. Two genes, *CYCLOIDEA* (*CYC*) and *DICHOTOMA* (*DICH*), belonging to the CYC/TB1 clade of the TCP transcription factor family, were found to promote the dorsal identity of zygomorphic flowers [11,12,13,14]. *RADIALIS* (*RAD*), a member of the MYB gene family, was found to be the downstream target of *CYC* and *DICH* [15,16,17]. Plants of the double *cyc*/*dich* or the single *rad* mutants produce flowers that have entirely or partially lost their dorsal identity [11,16]. The dorsal petals assume the ventral petal identity and the aborted dorsal stamen becomes functional [11]. *DIVARICATA* (*DIV*), a member of a different MYB lineage, promotes ventral floral identity [18]. A single *div* mutant causes the loss of the ventral petal identity [18,19]. In the *cyc*/*dich*/*div* triple mutant, where the function of both the dorsal and ventral identity genes was lost, all petals resume the lateral petal identity [18,19].

Recently, antagonism involving three MYB-like proteins was found to be a mechanism regulating floral symmetry in the flowers of *Antirrhinum* [10]. Despite the role of *DIV* in controlling ventral petal identity, its mRNA is transcribed across the floral meristem [18,19]. RAD was found to be the dorsal factor inactivating DIV, but not at the transcriptional level [10,19]. Interestingly, it was found that RAD and DIV do not directly interact with each other, but compete for their protein target, DIV-and-RAD-interacting-factors (DRIFs), also members of the MYB family [10]. In particular, DIV and DRIFs show overlapping expression patterns and can form heterodimer complexes that bind to the DNA of *DIV*, suggesting the regulation of its transcription. RAD inhibits the interaction between DIV and DRIFs in the dorsal regions of the flowers of *Antirrhinum* by either binding directly to a DRIF protein in the nucleus or/and by sequestering the DRIF proteins in the cytoplasm [10]. Therefore, RAD acts as the antagonist that blocks the binding of DIV, the agonist, with the DRIFs, which is required for regulating ventral symmetry in the flowers of *Antirrhinum*.

Similar antagonistic relationships involving three MYB homologs were reported in the fruit development of *Solanum lycopersicum* L. [20]. The fruit SANT/MYB binding protein1 (FSB1), a DRIF homolog, was found to form a protein complex with the transcription factor MYBI, a DIV homolog. The fruit SANT/MYB-like (FSM1) protein, a RAD homolog, competes for FSB1 with MYBI. The function of FSM1 is to reduce fruit size and preferentially restrict differential cell expansion [20]. Ectopic expression of FSM1 results in a reduction in organ size by negatively affecting cell expansion. In contrast, FSB1 positively regulates differential cell expansion through a physical interaction with MYBI [20]. This is analogous with the competition between RAD and the DIV-DRIF complex in the dorsal regions of the flowers of *Antirrhinum*. The function for the FSM1–FSB1–MYBI complex in tomato controls cell expansion, while RAD–DRIF–DIV similarly also controls cell expansion by regulating dorsoventral flower asymmetry in snapdragon [10,20].

Previous works indicated frequent gene duplications during the evolution of *RAD*- and *DIV*-like genes [21,22]. Three paralogs of the RAD lineage, RAD1, RAD2, and RAD3, as well as three paralogs of the DIV lineage, DIV1, DIV2, and DIV3, are recognized [21,22]. The gene duplications that gave rise to these paralogs were predicted to have occurred around the diversification of the Pentapetalae. Therefore, there may exist antagonistic relationships among the homologs of RAD–DRIF–DIV in diverse lineages of the core eudicots. DRIFs, one of the three factors involved in this antagonistic interaction, belong to an ancient MYB-like gene family with several homologs also found in the moss *Physcomitrella patens* [10]. Two paralogs of DRIFs resulting from gene duplication at least in the common ancestor of monocots and dicots are named Group 1 and 2 [10]. The DRIF1 and DRIF2 of *An. majus* belong to Group 1, while the only DRIF-like protein (SlFSB1) found in *So. lycopersicum* belongs to Group 2. Therefore, in the antagonized systems in *An. majus* and *So. lycopersicum*, the DRIF homologs involved belong to two paralogous clades.

Here, we report on the evolution of the I-box-like and R-R-type lineages where *RAD*- and *DIV*-like genes belong, respectively, and aim to (1) reconstruct the phylogeny of the two MYB lineages using the maximum likelihood (ML) and Bayesian algorithms, (2) clarify the phylogenetic relationships of the paralogs, and (3) identify the homology of *RAD*- and *DIV*-like genes that form the antagonistic relationships in *An. majus* and *So. lycopersicum*. We also focus on *RAD*-like gene evolution in Solanaceae, where lineage-specific gene duplications were identified. We demonstrate that, among similar antagonistic interactions in *An. Majus* and *So. lycopersicum*, *RAD*-like genes originate from the closely related ortholog, while *DIV*-like genes originate from distantly related paralogs. Furthermore, the phylogenies of the I-box-like and R-R-type lineages generated in this study will guide future works in understanding the functional divergence of these MYB lineages.

## 2. Results

### 2.1. RAD-Like Genes from Solanaceae

Sixteen sequences of *RAD*-like genes were discovered in this study (GenBank numbers MF398572-MF398587) (Table 1). We show that our cloning method can recover all of the RAD2 paralogs identified from the genome data of *P. hybrida* and *So. lycopersicum* (Table 1).

### 2.2. Diversity and Phylogeny of I-Box-Like MYB Genes

A total of 274 *RAD-*like coding DNA sequences (CDSs) were found in 101 species representing 28 families and 15 orders of dicots (Solanales, Vitales, Brassicales, Malvales, Malpighiales, Ranunculales, Lamiales, Saxifragales, Rosales, Fabales, Proteales, Cucurbitales, Myrtales, Dipsacales, and Sapindales) and monocots (Table S1). Among these sequences, 79 CDSs belong to 17 species of seven genera of Solanaceae, which includes the FSM1 from *So. lycopersicum* [20]. For *Arabidopsis*, six *RAD* homologs, At4g39250 (*Arabidopsis_thaliana_*RL1, NM_120086.2), At2g21650 (*Arabidopsis_thaliana_*RL2, NM_127736.3), At4g36570 (*Arabidopsis_thaliana_*RL3, BT011255.1), DQ395345 (*Arabidopsis_thaliana_*RL4, NM_001084443.1), At1g19510 (*Arabidopsis_thaliana_*RL5, NM_101808.4), and At1g75250 (*Arabidopsis_thaliana_*RL6, NM_001084356.2), were included.

**Figure 1:**
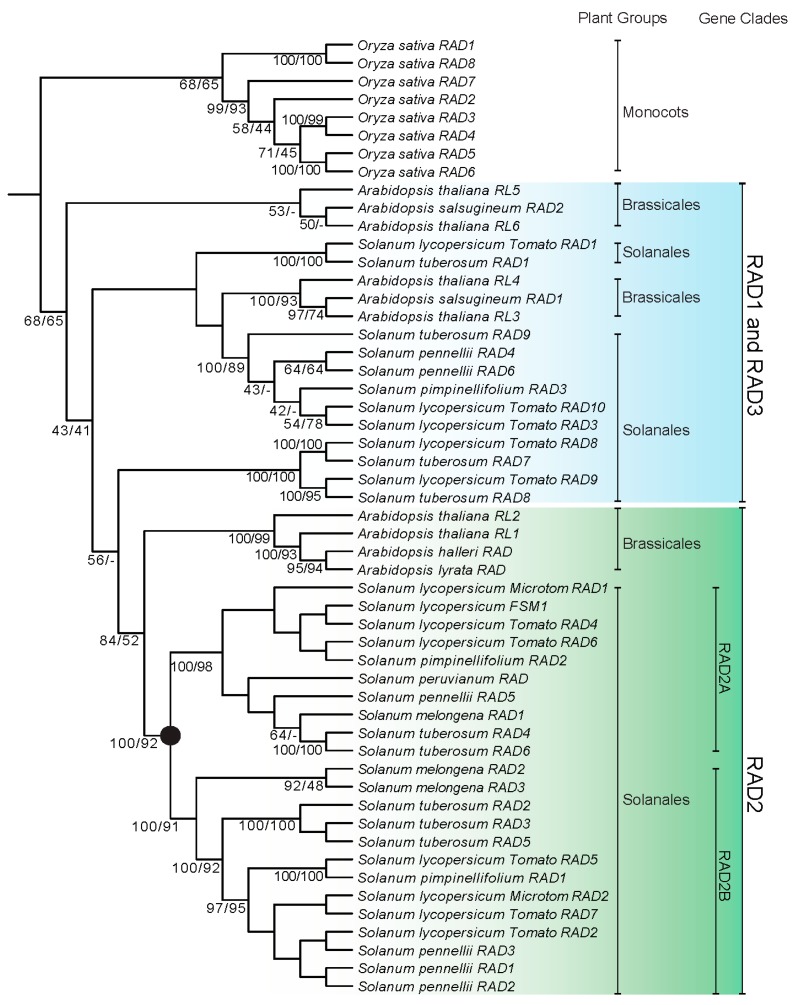
Phylogeny of *I-box-binding/RADIALIS-*like genes of four species of *Arabidopsis*, six species of *Solanum*, and *Oryza sativa* based on Bayesian and maximum likelihood (ML) inferences. All sequences from *O. sativa* formed a monophyletic clade was used to root the phylogeny. Based on the clade defined by Boyden et al. (2012), [21], only the RAD2 clade was monophyletic and contained sequences from *Arabidopsis* and *Solanum*. There are two paralogs in the RAD2 clade, i.e., RAD2A and RAD2B, which resulted from a gene duplication that *Arabidopsis* was not involved. RAD1 and RAD3 are paraphyletic. Bayesian posterior probabilities and bootstrap frequencies ≥40% depicted close to the branches, respectively.

A phylogeny of *RAD*-like genes was constructed based on 53 sequences from four species of *Arabidopsis* (*A. thaliana*, *A. halleri*, *A. lyrata*, and *A. salsuginea*), six species of *Solanum* (*So. melongena So. pennellii*, *So. lycopersicum*, *So. pimpinellifolium*, *So. peruvianum*, and *So. tuberosum*), and *Oryza sativa* (Figure 1). The phylogeny indicated that sequences from *O. sativa* form a monophyletic clade. However, the phylogenetic relationships among the three previously identified RAD1, RAD2, and RAD3 clades [21] were not fully resolved. The RAD2 clade is likely monophyletic while the RAD1 and RAD3 clades are not (Figure 1, also see below). The RAD2 clade consists of *Arabidopsis thaliana* RL1 and *Arabidopsis thaliana* RL2 and species of *Solanum*, which were further divided into two *Solanum*-specific clades, RAD2A and RAD2B. The *FSM1* of *So. lycopersicum* was placed in the RAD2A clade. It is unclear, however, how the other sequences of *Solanum* should be placed within the RAD1 clade represented by *Arabidopsis thaliana* RL3 and *Arabidopsis thaliana* RL4 and with the RAD3 clade represented by *Arabidopsis thaliana* RL5 and *Arabidopsis thaliana* RL6 (Figure 1) [21].

**Figure 2:**
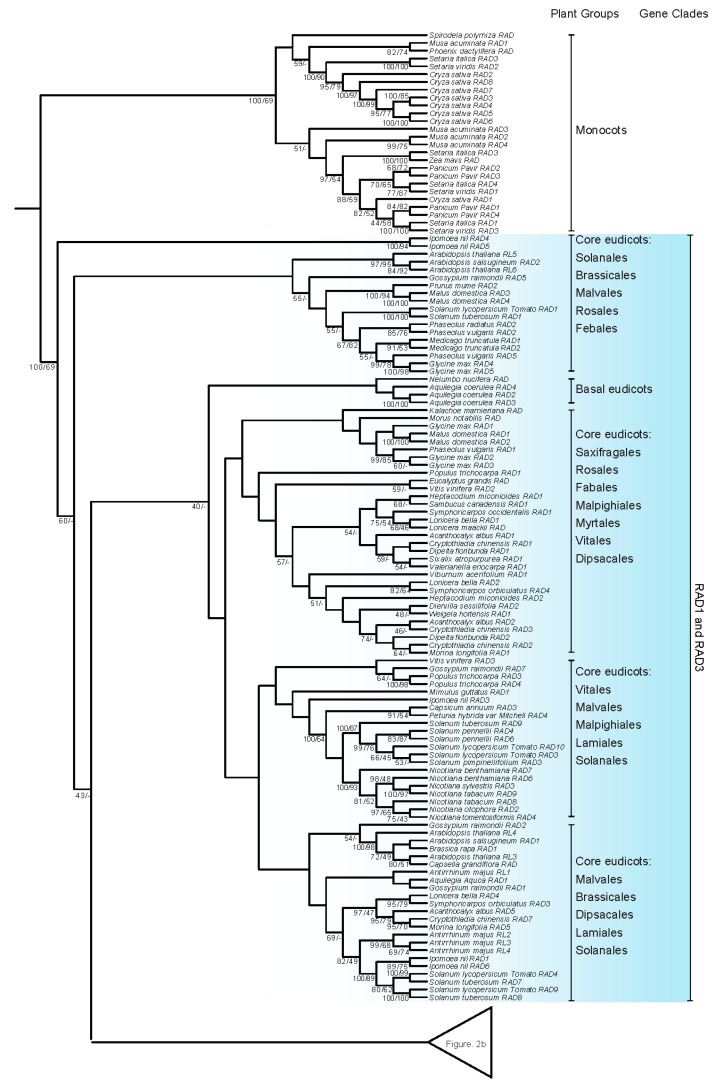

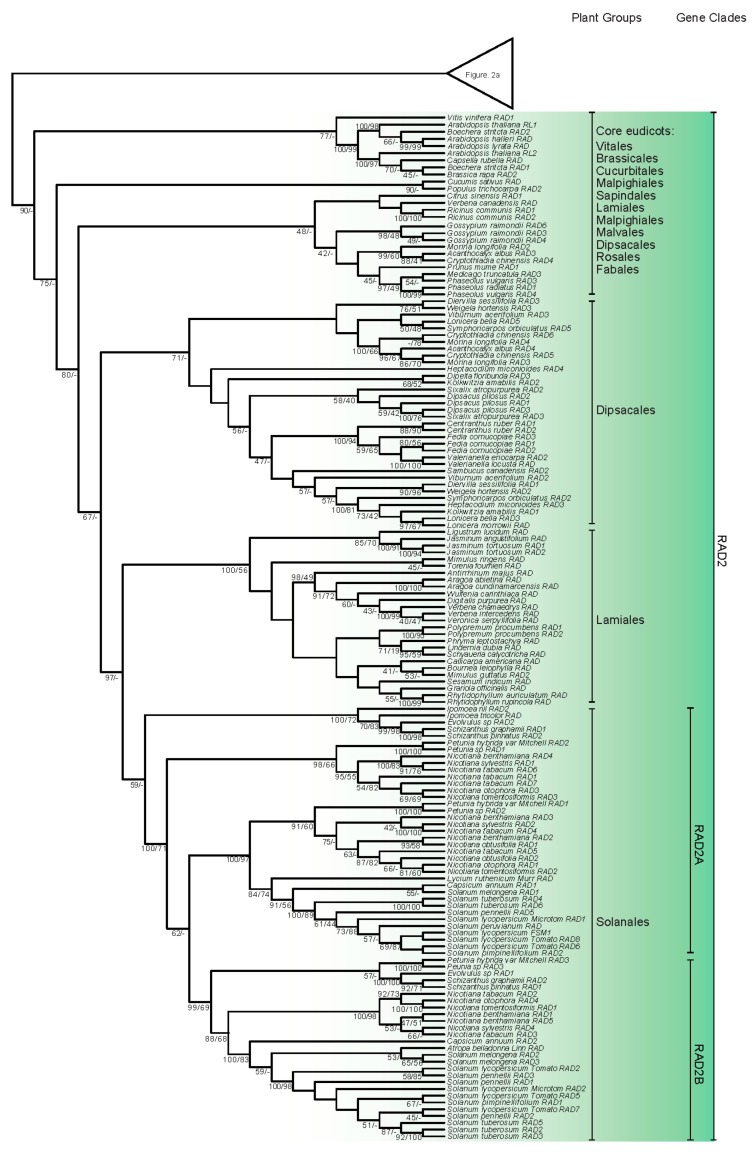
Phylogeny of *I-box-binding/RADIALIS-*like genes based on Bayesian and ML inferences. 274 CDSs of *I-box-binding/RADIALIS-*like genes from both monocots and dicots were analyzed. All sequences from monocots formed a monophyletic group was used to root the phylogeny. RAD2 formed a monophyletic clade. At least one gene duplication was identified in the common ancestor of Solanaceae and Convolvulaceae. RAD1 and RAD3 clades are paraphyletic. Bayesian posterior probabilities and bootstrap frequencies ≥40% depicted close to the branches, respectively.

Another phylogeny of *RAD-*like genes was reconstructed based on 274 CDSs, including 258 from blast results and 16 in this study (Figure 2, Table S1). All eight species from seven families of monocots form a monophyletic clade and were used to root the phylogeny. RAD2 forms a monophyletic clade, while both RAD1 and RAD3 were not fully resolved (Figure 1 and Figure 2). RAD2 comprises representatives from eleven orders: Vitales, Rosales, Malvales, Fabales, Cucurbitales, Sapindales, Malpighiales, Brassicales, Solanales, Lamiales, and Dipsacales (Figure 2 and Table S1). Most of the solanaceous and convolvulaceous *RAD*-like sequences fell into the RAD2 clade, which is further divided into two clades, RAD2A and RAD2B (Figure 1 and Figure 2; Figure S1). The unrooted phylogeny including only RAD2 of Solanaceae and Convolvulaceae further indicates that two paralogs have been likely formed at least in the common ancestor of the two families (Figure S1). Further gene duplication and gene losses likely also occurred, which led to *Nicotiana* and *Petunia* having additional paralogs in RAD2A (Figure 2, Figure S1). RAD2 sequences from the two species of *Schizanthus*, the first branching clade of Solanaceae [23], are more closely related to the sequences from Convolvulaceae, which might be due to the limited sampling. The *FSM1* of *So. lycopersicum* expressed in fruit is grouped in the RAD2A clade, while the *RAD* of *A. majus* is also in the RAD2 clade.

### 2.3. Diversity and Phylogeny of R-R-Type MYB Genes

One thousand and seventy-five CDSs that represent both *R-R-*type and *CCA1*-like genes from 109 species representing 34 different families from 22 orders of plants (16 of dicots, four of monocots, and two of mosses) were recovered (Table S2). For *A. thaliana*, the BLASTn results nine *R-R-*type, i.e., At1g49010 (AY519528.1), At2g38090 (AY519529.1), At3g11280 (AY550308.1), At5g01200 (AY519530.1), At5g05790 (AY519531.1), At5g08520 (AY519532.1), At5g58900 (AY519533.1), At5g23650 (DQ056685.1), and At5g04760 (AB493736.1) and one *CCA1*-like gene, i.e., At3g16350 (AY519512.1) [24]. For Solanaceae, we recovered 124 CDSs, namely *DIV-*, *MYB-* or *MYB1R1*-like genes, from 12 species in four genera, including the *MYBI* of *So. lycopersicum*.

**Figure 3:**
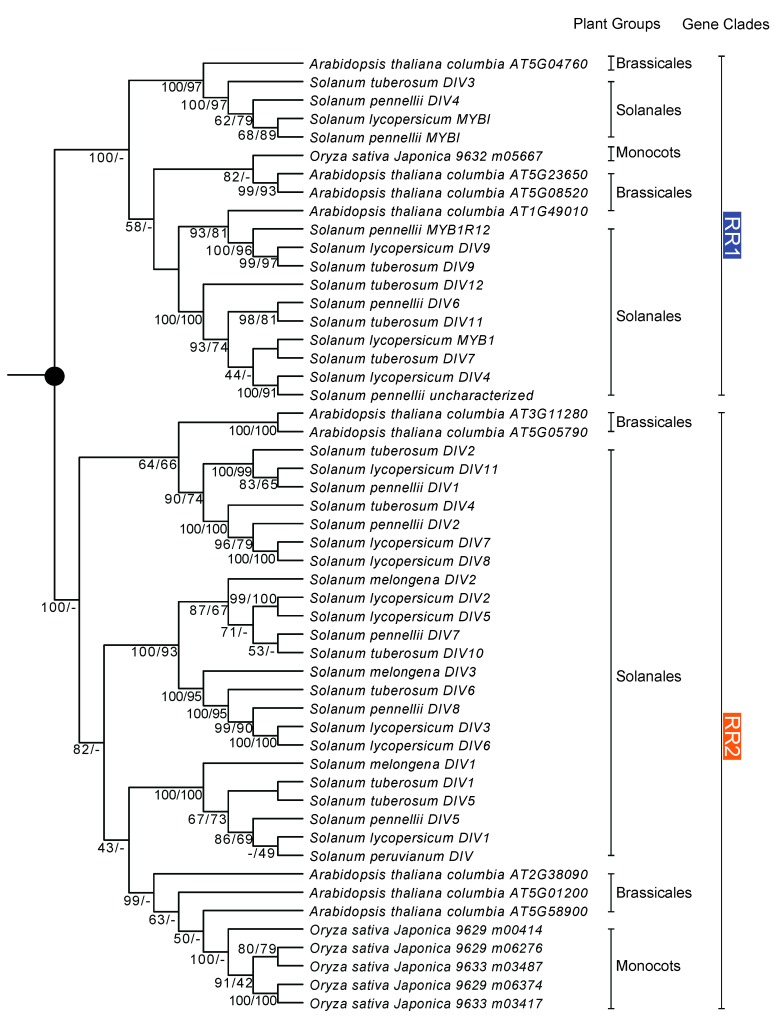
Phylogeny of *R-R-*type genes of five species of *Solanum*, *Arabidopsis thaliana*, and *Oryza sativa* based on Bayesian and ML inferences. Two major clades, RR1 and RR2, were identified, each of which includes sequences from *Arabidopsis*, *Oryza*, and *Solanum*. Bayesian posterior probabilities and bootstrap frequencies ≥40% depicted close to the branches, respectively.

An *R-R-*type gene phylogeny was first reconstructed based on 52 CDSs from *O. sativa japonica*, *A. thaliana*, and five species of *Solanum* (*So. melongena*, *So. lycopersicum*, *So. pennellii*, *So. peruvianum*, and *So. tuberosum*) (Figure 3). All sequences fell into two clades, RR1 and RR2/DIV. The RR2/DIV clade represented the DIV clade identified by Howarth and Donoghue [22]. Each of these two clades contained sequences from *O. sativa*, *A. thaliana*, and *Solanum*.

**Figure 4:**
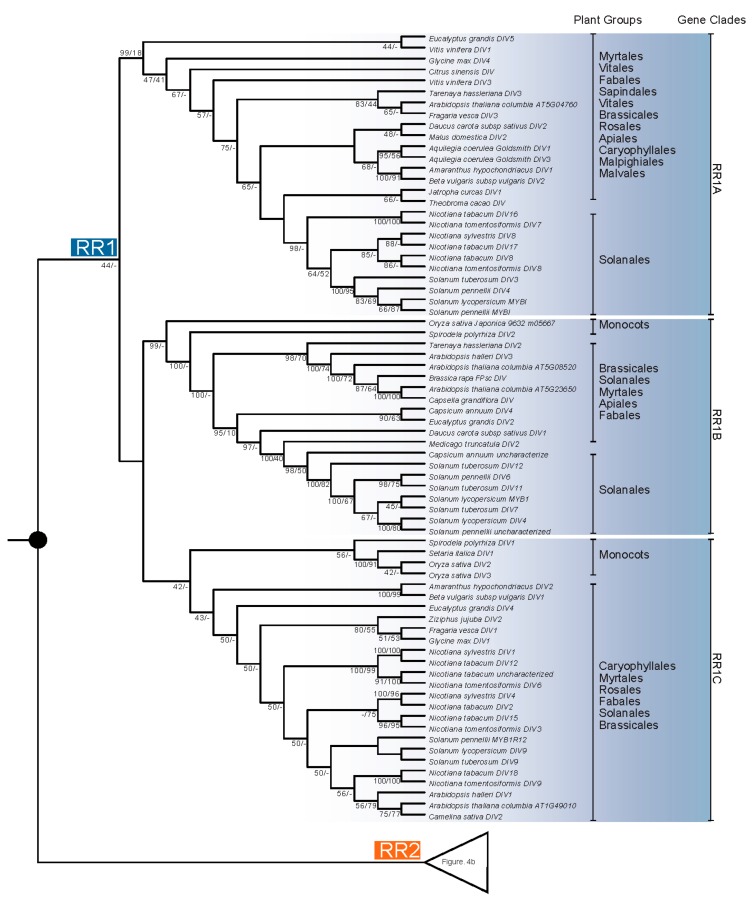

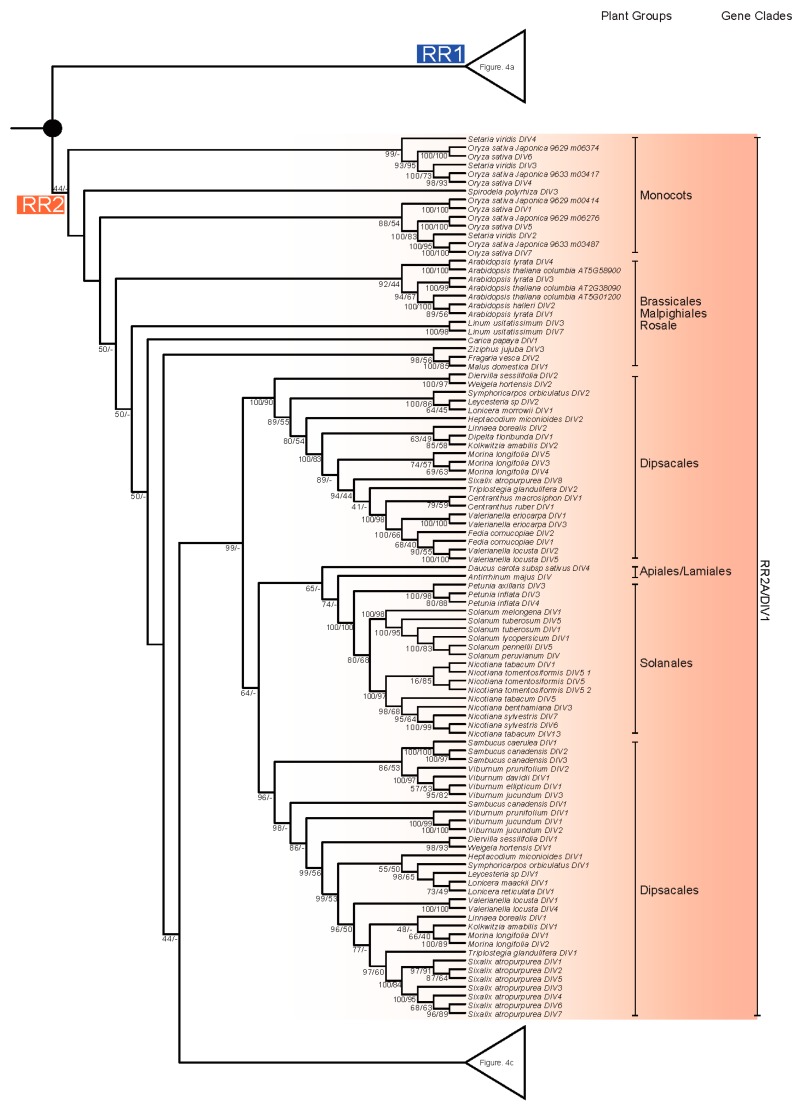

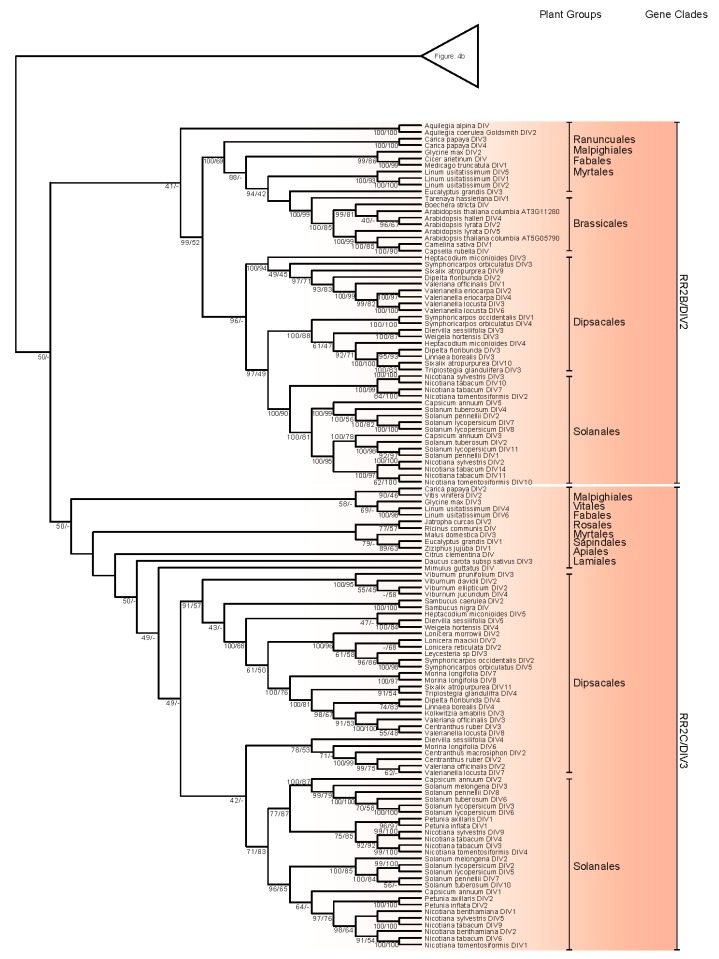
Phylogeny of *R-R-*type genes based on Bayesian and ML inferences. Two hundred and ninety-eight CDSs of *R-R-*type genes from both monocots and dicots were analyzed. They formed two major clades, RR1 and RR2/DIV, each of which contained sequences from monocots and dicots. The RR1 clade was further divided into three groups, RR1A, RR1B, and RR1C. For the three RR2/DIV clades identified by Howarth and Donoghue [22] only the DIV2 and DIV3 are monophyletic. The *Arabidopsis* sequences include AT2G38090, AT5G01200, and AT5G58900 identified as DIV1, which is not a clade in this phylogeny. Bayesian posterior probabilities and bootstrap frequencies ≥40% depicted close to the branches, respectively.

The *R-R*-type gene phylogeny was also reconstructed based on 298 CDSs from 75 species of 23 families (Figure 4, Table S2). The unrooted tree indicated that the RR1 and RR2/DIV clades were monophyletic (Figure 4). RR1 was further divided into three clades i.e., RR1A, RR1B, and RR1C. The RR1A clade included sequences from 12 orders of dicots (Myrtales, Fabales, Sapindales, Vitales, Brassicales, Rosales, Malvales, Malpighiales, Ranunculales, Caryophyllales, Apiales, and Solanales). The RR1B clade had representatives from monocots and five orders of dicots (Myrtales, Brassicales, Fabales, Apiales, and Solanales). RR1C clade had representatives from monocots and six orders of dicots (Caryophyllales, Myrtales, Brassicales, Rosales, Fabales, and Solanales). For *Arabidopsis*, AT5g04760 was placed in the RR1A clade, AT5G08520 and At5g23650 in the RR1B clade, and AT1G49010 in the RR1C clade. For the RR2/DIV clade, previously identified DIV2 and DIV3 clades formed monophyletic clades [22]. The sequences of *A. thaliana*, At2g38090, At5g01200, At5g58900, belonged to DIV1, while At3g11280 and At5g05790 belonged to DIV2. *Arabidopsis* lacked the *DIV3* copy based on previous work [22]. The *MYBI* of *So. lycopersicum* expressed in fruit was grouped in the RR1A of RR1 clade, while the *DIV* of *A. majus* was likely in RR2A/DIV1 of the RR2 clade.

### 2.4. Testing the Tree Topology for R-R-Type Genes

We further examined whether either of the two clades of *R-R*-type genes, RR1 including RR1A, RR1B, and RR1C; and RR2 including RR2A, RR2B, and RR2C, are monophyletic. Our results indicate that the tree topology number one, of which the subclades RR1A, RR1B, and RR1C formed a monophyletic RR1 clade, and the subclades RR2A, RR2B, and RR2C formed a monophyletic RR2 clade, is the most likely phylogeny [*p* > 0.5; Kishino-Hasegawa test (KH) = 0.794, Shimodaira-Hasegawa test (SH) = 0.997, and Approximately Unbiased test (AU) = 0.872] (Figure S2, Table 2). All the other tree topologies, except for the tree topology number nine, which have the RR2A subclade grouped within the RR1 clade, were rejected. However, the tree topology number nine is not strongly supported (0.2 < *p* < 0.5; KH = 0.206, SH = 0.343, and AU = 0.213) compared to the tree topology number one (Figure S2, Table 2).

### 2.5. Motif Analyses

**Figure 5:**
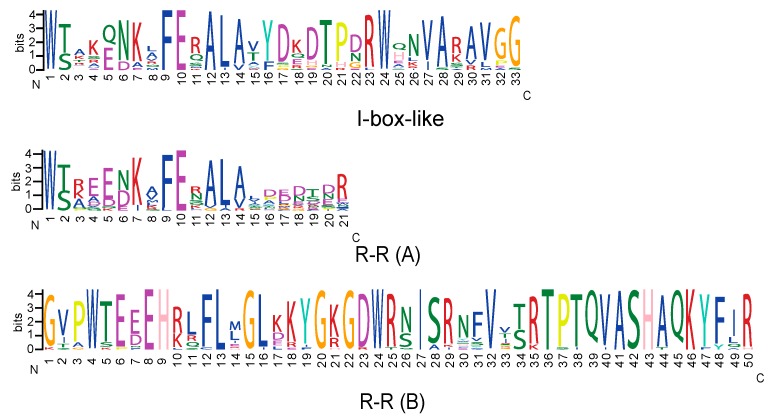
Motif analyses of *I-box*-like and *R-R*-type genes of the MYB family. One motif was identified for the *I-box*-like genes and two motifs, i.e., R-R (**A**) and R-R (**B**), were found for the *R-R*-type genes. The x-axis indicates the sequence of the amino acids from the N- to C- terminal for each motif; the y-axis provides the information at each position in the motif; and the highly conserved positions in the motif show a large proportion of the bits for a particular amino acid.

We also analyzed the nucleotide sequences, which cover the diverse lineages of the I-box-like and R-R-type from *A. thaliana* and *O. sativa*, and the representatives from *An. majus* and *So. lycopersicum*, to identify protein motifs. Our results largely agree with the study of Chen et al. [24], which analyzed the motifs for the *I-box*-like, *R-R*-type, and *CCA1*-like MYB genes. We found that the *I-box-*like genes have only one motif, while the *R-R-*type genes have two motifs, i.e., R-R (A), and R-R (B). R-R (A) locates at the N-terminal of the *R-R-*type genes, and shows high similarity to the *I-box*-like genes (Figure 5). R-R (B) locates at the C-terminal of the *R-R-*type genes, and is distinct in amino acid sequences compared to the R-R (A) and the only motif of *I-box*-like genes.

On the other hand, the results of our motif analyses also show differences compared to the results of Chen et al. [24]. For *I-box*-like genes, our analyses identified a single motif that is 33 amino acids in length. In contrast, the same motif based on Chen et al. [24] contains 56 amino acids, which has eight and 15 extra amino acids at the N- and C-terminal, respectively. For *R-R*-type genes, our results indicate that R-R (A) is 21 amino acids in length, while Chen′s results [24] include 59 amino acids for the same motif, which has eight and 30 extra amino acids at the N- and C-terminal, respectively. Our results suggest that R-R (B) is 50 amino acids in length. However, the same motif based on the work of Chen et al. [24] has 53 amino acids, which has five extra amino acids at the N-terminal but lacks two amino acids at the C-terminal. One possible reason for the discrepancy between the two studies is that the Multiple Expectation maximizations for the Motif Elicitation (MEME) methods applied in our study only consider the continued amino acid sequences for a motif and no gap in the sequences is allowed.

## 3. Discussion

### 3.1. Phylogenetic Positions of RAD- and DIV-Like Genes in the Plant MYB Lineage.

MYB proteins contain a conserved MYB domain, which usually comprises one to three imperfect repeats, namely R1, R2, R3 [4,24]. Each of these repeats comprises about 52 amino acid residues that encode a helix-loop-helix structure involved in DNA binding [4,25]. *MYB* genes have been found in all eukaryotes [4,26].

Phylogenetic analysis indicates that the *MYB* genes of plants, which is sister to all animal *MYB* genes, form a monophyletic clade [25]. *MYB* genes in plants are structurally and functionally more variable compared to *MYB* genes in vertebrates [25,27]. Based on the MYB domain structures, the MYB proteins of plants can be classified into three major groups: R1R2R3-MYB with three adjacent repeats, R2R3-MYB with two adjacent repeats, and MYB-related proteins, a heterogeneous group, often containing a single MYB repeat [7,24,25,28,29,30]. The R2R3-MYB group is thought to be derived from the R1R2R3-MYB group, which occurs in all major lineages of land plants [27]. Based on the phylogenetic analysis and the protein domain structure, MYB-related proteins were further divided into five subfamilies: CCA1-like, CPC-like, TBP-like, I-box-binding-like (abbreviated I-box-like), and R-R-type [24,30]. Based on Chen et al. [24], *A. thaliana* has five *I-box*-like genes, i.e., At1g75250, At1g19510, At2g21650, At4g39250, and At4g36570, and nine *R-R*-type genes, i.e., At1g49010, At2g38090, At3g11280, At5g01200, At5g05790, At5g08520, At5g58900, At5g23650, and At5g04760. Boyden, Donoghue, and Howarth [21] indicated that *RAD-*like genes belong to the I-box-like clade. Our analyses further indicate that the I-box-like lineage is synonymous with *RAD*-like genes (Figure 1 and Figure 2). Furthermore, Howarth and Donoghue [22] focused on the evolution of *DIV*-like genes in core eudicots especially in Dipsacales, and indicated that the *DIV*-like genes belong to an R-R-type lineage. Our analysis of *R-R-*type genes showed that the gene duplication occurred at least in the common ancestor of dicots and monocots, giving rise to two paralogs, the RR1 and RR2 clades (Figure 3 and Figure 4), of which the RR2 clade is synonymous with the DIV-like lineage [22].

### 3.2. Evolution of the I-Box-Like Subfamily

Boyden, Donoghue, and Howarth [21] indicated that *RAD-*like genes consist of three major clades: RAD1, RAD2, and RAD3, which were speculated to result from genome duplications associated with the origin of core eudicots. The RAD1 clade has *Arabidopsis* AT4G36570 and DQ395345 of Clade I, defined in Reference [24], and RAD2 and RAD3 have the *Arabidopsis* sequences from Clade III (AT2G21650 and AT4G39250 belong to the RAD2, and AT1G19510 and AT1G75250 belong to RAD3). Our analysis recognized RAD2 as a monophyletic clade (Figure 1 and Figure 2). Furthermore, there are two RAD2 paralogs involving Solanaceae and Convolvulaceae, RAD2A and RAD2B, which likely resulted from a gene duplication at least in the common ancestor of these two plant families. On the other hand, the RAD1 and RAD3 clades were not fully resolved based on our analyses. Our phylogenetic analyses indicated that the *RAD* of *A. majus* belongs to the RAD2 clade, while *FSM1* is placed in the RAD2A clade, suggesting that *RAD* and *FSM1* belong to the same orthologous lineage.

### 3.3. Evolution of the R-R-Type Subfamily

The *R-R-*type genes have two imperfect repeats of the MYB domain, namely R-R (A) and R-R (B) [24]. The N-terminal MYB repeat R-R (A) was found to be closely related to the MYB repeats of the *I-box*-like genes, and the C-terminal MYB repeat R-R (B) was closely related to those of certain *CCA1-*like genes based on the positions of the introns and shared motifs [24]. The phylogeny of *R-R*-type genes based on nine sequences of *A. thaliana* and seven of *O. sativa japonica* suggests several gene duplications in the common ancestor of the monocots and dicots, but the phylogenetic relationships of the predicted paralogs were unresolved in that study [24]. The work by Howarth and Donoghue [22] focused on the evolution of *DIV*-like genes in core eudicots, especially in Dipsacales, which showed duplications giving rise to three DIV-like clades in the core eudicots, DIV1, DIV2, and DIV3. Our blast and phylogenetic analyses indicated that most of the sequences named *DIV*-like genes belong to the R-R-type subfamily, while most of the sequences named as *MYB1R1*-like genes belong to the *CCA1*-like gene family (Table S2). Each of the two R-R-type subclades, RR1 and RR2, was further divided into three paralogs, which likely resulted from genome duplication in the common ancestor of core eudicots [22]. RR1 consists of RR1A, RR1B, and RR1C, while RR2/DIV is composed of RR2A/DIV1, RR2B/DIV2, and RR2C/DIV3 (Figure 3 and Figure 4) [22]. We found that the *DIV* of *An. majus* belongs to the DIV1 of the RR2/DIV clade [22], while the *MYBI* of tomato belongs to the RR1A of the RR1 clade.

### 3.4. Evolution of the Antagonism among RAD-DRIF-DIV and FSM1-FSB1-MYBI in An. majus and So. lycopersicum, Respectively

Based on an analysis of amino acid sequences, the two MYB domains of DIV had different functions with the C-terminal domain similar to known DNA binding MYB proteins, while the N-terminal domain was associated with protein-protein interactions (Figure 5) [19,31]. In contrast, RAD has a single MYB domain that is predicted to act through a mechanism involving protein–protein interactions (Figure 5) [16]. As the members of MYB-related subfamilies, *I-box*-like and *R-R-*type genes were previously placed in the same clade by Riechmann and Ratcliffe [30], which suggested that they might be closely related paralogs. One possible hypothesis proposed for the evolution of these two MYB-related subfamilies is that *I-box*-like genes evolved through the loss of the MYB domain at the C-terminal end [24,32]. RAD-DRIF-DIV and FSM1-FSB1-MYBI therefore represent the recruitment of homologous genes from similar MYB lineages in the development of floral zygomorphy in *An. majus*, and the development of fruit in *So. lycopersicum* [10].

In summary, I-box-like and R-R-type lineages have experienced extensive gene duplication that predated the diversification of the core eudicots. Our work further clarified the evolution of these two MYB subfamilies, which will help the future inquiry into the functional studies of the paralogs of the *I-box*-like and *R-R*-type genes that may have been involved in the evolution of molecular antagonism.

## 4. Materials and Methods

### 4.1. Cloning RAD-Like Genes from Species of Solanaceae and Convolvulaceae

Primers incorporated with degenerate polymorphic sites based on the alignment of *RAD*-like sequences, especially the RAD2 clade from Solanaceae and Lamiales, were used for amplifying the genes from species of Solanaceae and representatives of Convolvulaceae. The locations of our primers referred to the study by Boyden, Donoghue, and Howarth [21]. These primers, i.e., forward primer 5′-AACAAGGCITTTGARARGGCWTYRGC-3′, and reverse primer 5′-GGRAARGGBAYIMYACCAIDITCAAT-3′, successfully amplified *RAD*-like genes from both the basal and derived clades of Solanaceae (*Schizanthus pinnatus* Ruiz & Pav, *Schizanthus grahamii* Gillies, *Petunia sp.*, *Nicotiana obtusifolia* M. Martens & Galeotti, *Solanum lycopersicum* L., *Lycium ruthenicum* Murray, and *Atropa belladonna* L.) and species of Convolvulaceae (*Evolvulus sp*. and *Ipomoea tricolor* Cav.) (Table 1). PCR reactions were performed using GoTaq^®^ G2 Hot Start Polymerase (Promega, Madison, WI, USA), as follows: 95 °C for 5 min, 95 °C for 45 s, 55 °C for 45 s, and 72 °C for 1 min and 30 s, repeated for 39 cycles, with a final step at 72 °C for 10 mins. PCR products were then purified through gel extraction using Wizard SV Gel and PCR Clean-Up System from Promega. The purified PCR products were used as a template for the second round of PCR following the same PCR program described above. The purified second round PCR products were used in ligation and transformation with pGEM-T Easy Vector System I from Promega. At least 50 clones were screened for each species. The sequences of the clones were determined using Sanger sequencing by GENEWIZ (115 Corporate Boulevard, South Plainfield, NJ, USA).

### 4.2. Gene Mining

The *RAD*- and *DIV*-like genes were obtained through blasting *RAD* and *DIV* CDSs of *A. majus* (GenBank accession numbers: AY954971.1 and AY077453.1, respectively) against the databases, including NCBI BLASTn (available online: http://www.ncbi.nlm.nih.gov/ BLAST/), Phytozome 11 (available online: https://phytozome.jgi.doe.gov), Sol Genomics Network (available online: https://solgenomics.net), and Rice Genome Annotation Project (available online: http://rice.plantbiology.msu.edu).

### 4.3. Alignment and Phylogenetic Analyses

The DNA matrices of the coding sequences were aligned using Geneious version 7.1.9 (PO Box 5677, Wellesley St, Auckland 1010, New Zealand). The MUSCLE algorism that refers to the protein sequence alignment for building nucleotide sequence alignment was applied. Each DNA matrix was analyzed by using the Bayesian and ML inferences, which were implemented in RAxML_HPC2, and MrBayes version 3.2.6 on XSEDE, respectively, at the CIPRES Science Gateway V. 3.3. [33,34,35,36]. For ML analyses, a random seed value for rapid ML bootstrapping was estimated on each dataset. The GTRCAT model was chosen for the bootstrapping analysis based on the program recommendation because GTRCAT shows lower computational costs and memory consumption for the ML method [34]. The models used for the Bayesian analyses were estimated using jmodeltest 2.1.10 [37,38]. The Akaike Information Criterion (AIC) [39] was used to determine the best-fit model for each DNA sequence matrix, i.e., K80 (K2P) + g model for the *I-box-*like/*RAD* gene phylogeny including *Arabidopsis*, *Solanum*, and *Oryza* alone, JC + g model for the large *RAD* phylogeny, GTR + i + g model for the *R-R-*type gene phylogeny including *Arabidopsis*, *Solanum*, and *Oryza* alone, and GTR + i + g model for the large *R-R-*type gene phylogeny. We used the Metropolis-coupled Markov chain Monte Carlo method as implemented in MrBayes to run four chains. We ran five million generations for each chain, and sampled every 1000 generations with a burn-in of the first 2000 trees.

### 4.4. Phylogeny Assessment for R-R-Type Genes

We generated 14 tree topologies manually based on our Bayesian tree to test the alternate hypotheses. To produce these topologies, first the RR2A clade was set as monophyletic. Second, we collapsed the relationships among the subclades in RR1 and RR2 clades. Finally, the subclades were subsequently moved around as indicated in Figure S2. The log-likelihoods for each tree topology were calculated using TREE-PUZZLE (ver. 5.3.rc16) [40] with the HKY model of evolution [41] and four rate categories for the discrete Gamma distribution. The log-likelihoods information estimated for the 14 topologies from TREE-PUZZLE was then entered into CONSEL (ver. 0.20) [42] to generate the bootstrap replicates for each tested tree. The *p-*values of KH [43], SH [44], and AU tests [45] were subsequently calculated based on the bootstrap samples [42]. The confidence of the 14 trees was then assessed by the *p-*values [42]. If the *p-*value estimated for a tree was < 0.05, the topology was rejected; if the *p* value >0.5, the topology was preferred [45,46].

### 4.5. Motif Analyses

Motif analyses based on nucleotide sequences were carried out for *I-box-*like and *R-R-*type genes. For *I-box-*like genes, we included six sequences of *A. thaliana*, i.e., At4g39250 (*Arabidopsis_thaliana_RL1*, NM_120086.2), At2g21650 (*Arabidopsis_thaliana_RL2*, NM_127736.3), At4g36570 (*Arabidopsis_thaliana_RL3*, BT011255.1), DQ395345 (*Arabidopsis_thaliana_RL4*, NM_001084443.1), At1g19510 (*Arabidopsis_thaliana_RL5*, NM_101808.4), and At1g75250 (*Arabidopsis_thaliana_RL6*, NM_001084356.2); eight sequences of *O. sativa*, i.e., (*Oryza_sativa_RAD1*, LOC_Os01g44390.2), 9640.m03280 (*Oryza_sativa_RAD2*, LOC_Os12g33950), 9631.m01422 (*Oryza_sativa_RAD3*, LOC_Os03g14810), 9631.m06332 (*Oryza_sativa_RAD4*, LOC_Os03g63890), 9633.m03415 (*Oryza_sativa_RAD5*, LOC_Os05g37040), 9633.m03416 (*Oryza_sativa_RAD6*, LOC_Os05g37050), 9635.m02514 (*Oryza_sativa_RAD7*, LOC_Os07g26150.1), and 9640.m03280 (*Oryza_sativa_RAD8*, LOC_Os12g33950); one sequence of *An. majus*, i.e., *RAD*; and one sequence of *So. Lycopersicum*, i.e., *FSM1.*

For *R-R-*type genes, we included nine sequences of *A. thaliana*, i.e., At1g49010 (AY519528.1), At2g38090 (AY519529.1), At3g11280 (AY550308.1), At5g01200 (AY519530.1), At5g05790 (AY519531.1), At5g08520 (AY519532.1), At5g58900 (AY519533.1), At5g23650 (DQ056685.1), and At5g04760 (AB493736.1); seven sequences of *O. sativa*, i.e., 9632.m05667 (LOC_Os04g58020), 9629.m00414 (LOC_Os01g04930), 9629.m06276 (LOC_Os01g63460), 9629.m06374 (LOC_Os01g64360), 9633.m03417 LOC_Os05g37060), 9633.m03487 (LOC_Os05g37730), and 9631.m06132 (LOC_Os03g62100); one sequence of *An. majus*, i.e., *DIV*; and one sequence of *So. lycopersicum*, i.e., *MYBI.*

The nucleotide sequences of these CDSs were translated into amino acid sequences by Mesquite version 3.2 [47]. We used the MEME algorithm, which extends the Expectation Maximization (EM) algorithm for identifying motifs in unaligned amino acid sequences [48]. The MEME algorithm is designed to discover novel and ungapped motifs in a set of homologous sequences [48]. To use this function, we uploaded and analyzed the *I-box*-like and *R-R*-type amino acid sequences at http://meme-suite.org/index.html [49]. For the MEME options, we set the numbers of motifs to be found as three, and each motif occurred only one time in each testing sequence.

## Figures and Tables

**Table 1:** Species sampled for the RAD2 clade with collection locations, voucher information, sequence name, phylogenetic placement, and number of clones sequenced.

Species	Family	Location	Voucher	Sequence Names	Clades	# of Clones Sequenced
*Petunia sp.*	Solanaceae	VCU Greenhouse	Zhang_Lab_23 (VCU)	*Petunia sp RAD1*	RAD2A	12
				*Petunia sp RAD2*	RAD2A	20
				*Petunia sp RAD3*	RAD2B	8
*Lycium ruthenicum* Murray.	Solanaceae	Taxkorgan Tajik Autonomous County, Xinjiang, China	CPG13183 (PE)	*Lycium ruthenicum Murr RAD*	RAD2A	20
*Atropa belladonna* L.	Solanaceae	Hotel Elites, Nathia Gali, Northwest Frontier Province, Pakistan	CPG13594 (PE)	*Atropa belladonna Linn RAD*	RAD2B	20
*Schizanthus pinnatus* Ruiz & Pav.	Solanaceae	VCU Greenhouse	Zhang_Lab_20 (VCU)	*Schizanthus pinnatus RAD1*	RAD2B	21
				*Schizanthus pinnatus RAD2*	RAD2A	22
*Schizanthus grahamii* Gillies	Solanaceae	VCU Greenhouse	Zhang_Lab_19 (VCU)	*Schizanthus grahamii RAD1*	RAD2A	21
				*Schizanthus grahamii RAD2*	RAD2B	19
*Nicotiana obtusifolia* M.Martens & Galeotti.	Solanaceae	VCU Greenhouse	Zhang_Lab_11 (VCU)	*Nicotiana obtusifolia RAD1*	RAD2A	14
				*Nicotiana obtusifolia RAD2*	RAD2A	16
*Solanum lycopersicum* L.	Solanaceae	VCU Greenhouse	Zhang_Lab_21 (VCU)	*Solanum lycopersicum microtom RAD1*	RAD2A	17
				*Solanum lycopersicum microtom RAD2*	RAD2B	13
*Evolvulus sp.*	Convolvulaceae	VCU Greenhouse	Zhang_Lab_18 (VCU)	*Evulupus sp RAD1*	RAD2B	20
				*Evulupus sp RAD2*	RAD2A	17
*Ipomoea tricolor* Cav.	Convolvulaceae	VCU Greenhouse	Zhang_Lab_22 (VCU)	*Ipomoea tricolor RAD1*	RAD2A	20

Virginia Commonwealth University (VCU) is in Richmond, VA, USA. VCU, Virginia Commonwealth University Herbaria; PE, Institute of Botany, Chinese Academy of Sciences Herbarium, Beijing, China.

**Table 2:** Comparison of the statistics of the phylogenetic hypotheses.

Tree Topology	*l*	*δ*	*p* Values		
			KH	SH	AU
1: [A-B-C] [D-E-F]	−34754.03	0.00	0.794	0.997	0.872
2: [B-C] [A-D-E-F]	−34775.47	21.45	0.037 *	0.284	0.048 *
3: [A-C] [B-D-E-F]	−34801.25	47.22	<0.001 *	0.029	<0.001 *
4: [A-B] [C-D-E-F]	−34808.97	54.95	<0.001 *	0.014	<0.001 *
5: [A] [B-C-D-E-F]	−34820.49	66.46	<0.001 *	0.003	<0.001 *
6: [B] [A-C-D-E-F]	−34820.49	66.46	<0.001 *	0.003	<0.001 *
7: [C] [A-B-D-E-F]	−34820.49	66.46	<0.001 *	0.003	<0.001 *
8: [A-B-C-D-E-F]	−34820.49	66.46	<0.001 *	0.003	<0.001 *
9: [A-B-C-D] [E-F]	−34774.05	20.02	0.206	0.343	0.213
10: [A-B-C-E] [D-F]	−34818.22	64.19	<0.001 *	0.004	<0.001 *
11: [A-B-C-F] [D-E]	−34818.03	64.00	<0.001 *	0.007	<0.001 *
12: [A-B-C-D-E] [F]	−34820.49	66.46	<0.001 *	0.003	<0.001 *
13: [A-B-C-D-F] [E]	−34820.49	66.46	<0.001 *	0.003	<0.001 *
14: [A-B-C-E-F] [D]	−34820.49	66.46	<0.001 *	0.003	<0.001 *

A = RR1A, B = RR1B, C = RR1C, D = RR2A, E = RR2B, and F = RR2C. *l* = Log-likelihood scores. *δ* = the log-likelihood differences to the best tree. * denotes statistical significance at the 0.05 level.

## Supplementary Materials

Supplementary materials can be found at www.mdpi.com/1422-0067/18/9/1961/s1.
