# Supplementary material for: Evolution of RAD- and DIV-Like Genes in Plants
Source: Int J Mol Sci. 2017 Sep 13;18(9):1961. doi: 10.3390/ijms18091961 (PMC5618610; doi:10.3390/ijms18091961)
Supplement: Supplementary file 1 [file ijms-18-01961-s001.zip › ijms-223486 supplementary - Figures.pdf]

Supplementary Materials: Evolution of *RAD*- and *DIV*-like Genes in Plants

Ao Gao, Jingbo Zhang and Wenheng Zhang *

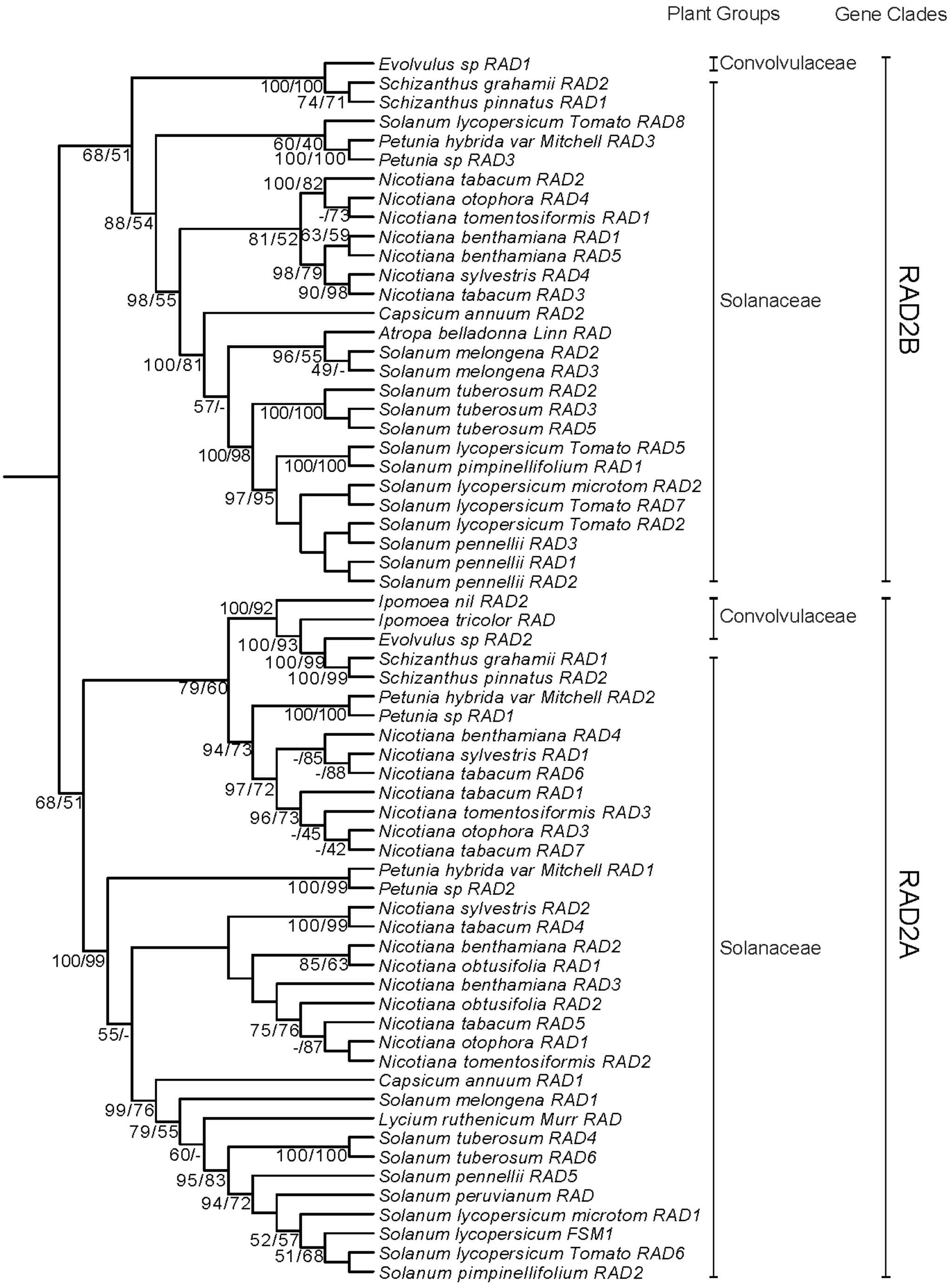

Figure S1. Phylogeny of RAD2 clade of Solanaceae and Convolvulaceae based on Bayesian and ML inferences. The unrooted tree shows the two clades, each of which includes sequences from both Solanaceae and Convolvulaceae. Bayesian posterior probabilities and bootstrap frequencies depicted close to the branches, respectively.

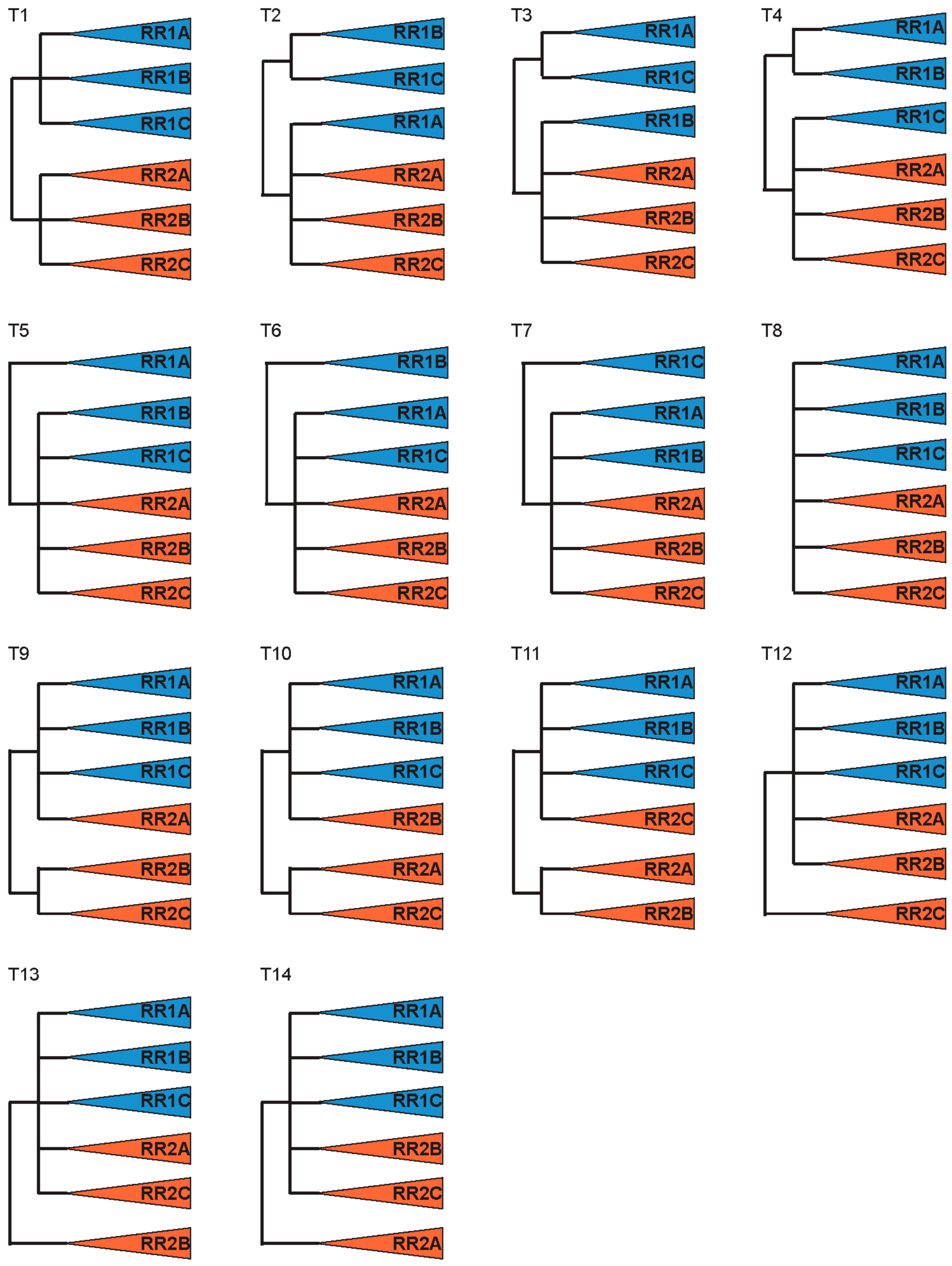

Figure S2. Illustration of the 14 alternative tree topologies that were statistically evaluated. The three subclades under tests within RR1 and RR2 clades are labeled with blue and orange, respectively. The number of each alternative topology in this figure matches with the number in Table 2.
